# The ubiquitin ligase CBL and Fas-associated factor 2 cooperate to regulate the innate immune response to *M. tuberculosis*

**DOI:** 10.1371/journal.ppat.1013974

**Published:** 2026-03-17

**Authors:** Tina Truong, Abigail Ray, Kelsey Martin, Nicholas A. Bates, Michelle Salemi, Brett S. Phinney, Bennett H. Penn

**Affiliations:** 1 Department of Internal Medicine, Division of Infectious Diseases, University of California, Davis, Sacramento, California, United States of America; 2 Graduate Group in Immunology, University of California, Davis, California, United States of America; 3 Microbiology Graduate Group, University of California, Davis, California, United States of America; 4 Proteomics Core Facility, University of California, Davis, California, United States of America; 5 Department of Medical Microbiology and Immunology, University of California, Davis, California, United States of America; University of Massachusetts Medical School, UNITED STATES OF AMERICA

## Abstract

As a first line of host defense, macrophages must be able to effectively sense and respond to diverse types of pathogens, and while a particular response may be beneficial in some circumstances, it can be detrimental in others. Upon infection, *Mycobacterium tuberculosis* (*Mtb*) induces proinflammatory cytokines and activates antibacterial responses. Surprisingly, *Mtb* also triggers antiviral responses that actually hinder the ability of macrophages to restrict *Mtb* growth. In *Mtb*-infected macrophages, the ubiquitin ligase CBL suppresses antiviral responses and preserves the antibacterial capacity of the macrophage. However, the mechanisms by which CBL regulates immune signaling are unknown. We found that CBL controls responses to multiple immune stimuli and broadly suppresses the expression of antiviral response genes. We used mass spectrometry to identify potential CBL substrates, and found, in total, over 46,000 ubiquitylated peptides in *Mtb*-infected macrophages, including roughly 400 peptides with CBL-dependent ubiquitylation. We then performed genetic interaction analysis of CBL and its putative substrates, and identified the Fas-associated factor 2 (FAF2) adapter protein as a key signaling molecule downstream of CBL. Together, these analyses reveal thousands of previously uncharacterized ubiquitin-mediated signaling events and identify an important new regulator of immune signaling.

## Author summary

Macrophages are immune cells that detect and integrate multiple microbial signals to mount an appropriate defense. During infection with *Mycobacterium tuberculosis* (Mtb), the bacterium that causes tuberculosis, macrophages are flooded with diverse stimuli that must be integrated into a coherent immune response. Some molecules released by *Mtb*, like cytosolic nucleic acids, are known to activate antiviral responses that can actually interfere with the macrophage’s antibacterial activity. Previous studies have implicated a ubiquitin ligase, CBL, in suppressing this detrimental antiviral program to preserve macrophage antibacterial activity during *Mtb* infection. However, the underlying mechanism and relevant targets are unclear. Here, we define mechanisms used by CBL to regulate the innate immune response during *Mtb* infection. Using proteomics, we profiled ubiquitin signaling during *Mtb* infection and found that CBL modifies hundreds of host proteins, including the protein FAF2. FAF2 decreases localization of toxic lysosomes to *Mtb*, thus promoting bacterial growth. CBL restrains FAF2 activity, enabling macrophages to traffic *Mtb* to lysosomes for killing. Together, these results identify a CBL–FAF2 pathway that helps macrophages balance competing antiviral and antibacterial programs and highlight CBL-dependent control points.

## Introduction

Macrophages must be able to effectively sense and respond to diverse types of pathogens, ranging from multicellular eukaryotic parasites to bacteria and viruses. Longstanding clinical observations have suggested potential antagonism between immune responses targeting different classes of microbes. For example, patients with a preceding viral infection carry roughly a 100-fold higher near-term risk of developing bacterial pneumonia from *Streptococcus pneumoniae* [1]. Similarly, while CD4 T helper 2 (Th2) cells are critical for responses to multicellular parasites, patients with Th2-dominant immune responses in *Mycobacterium leprae* lesions harbor higher bacterial loads and experience more severe disease [2].

Even in the simple scenario of a single immune cell encountering a lone pathogen, the mechanisms by which the type of pathogen is discerned, and an appropriate response mounted, remains poorly understood. Dozens of individual sensors for diverse bacterial, fungal, and viral pathogen-associated molecular patterns (PAMPs) have been described [3,4]. However, most pathogens, including *M. tuberculosis* (*Mtb*)*,* activate multiple sensors simultaneously. How host cells integrate multiple, sometimes discordant, pathogen-associated signals into a coherent, pathogen-appropriate, response remains unclear.

*Mtb* persists as a threat to human health. It currently infects roughly one fourth of the world’s population, causing a chronic, often lifelong infection, and tuberculosis (TB) kills an estimated 1–2 million people annually [5]. Macrophages infected with *Mtb* are immediately exposed to an array of cell wall-derived PAMPs such as lipoproteins, peptidoglycan and trehalose 6,6’-dimycolate, which activate cognate immune sensors such as TLR2, NOD2 and CLEC4E [6–15]. Many of these signaling pathways ultimately result in activation of nuclear factor kappa B (NF-κB) family transcription factors and the upregulation of proinflammatory cytokines such as TNF and IL1B, which mediate potent antibacterial responses that are critical for controlling *Mtb* replication [16–23].

Surprisingly, *Mtb* also activates numerous antiviral effectors in infected macrophages. *Mtb* rapidly permeabilizes its phagosome and releases bacterial nucleic acids into the host cytosol through unclear mechanisms [24–26], and this DNA activates several sensors, including cyclic GMP-AMP synthase (cGAS). cGAS then triggers a cascading activation of STING1, TBK1 and the transcription factors IRF3 and IRF7 that activate the expression of numerous genes, including type I interferons such as interferon beta (IFN-β) [27]. IFN-β then signals in an autocrine and paracrine manner to induce the expression of an additional set of interferon-stimulated genes (ISGs) [24,25,27–31]. Bacterial RNA is also introduced into the host cell, initiating signaling through the retinoic acid-inducible gene I (RIG-I) sensor, and promoting a sustained activation of IRF3/IRF7 [25]. While these initial observations came from mouse models, gene expression analysis of TB patients has detected similar activation of type I IFN in the peripheral blood of patients with active TB [32–34].

Several lines of evidence demonstrate that these antiviral responses interfere with the antibacterial capacity of macrophages. C3H-derived strains of mice harbor a polymorphism causing loss of the SP140 transcriptional repressor, resulting in hyperactivation of IFN-β. These strains are highly susceptible to *Mtb*, succumbing rapidly with dramatically increased bacterial burdens [35–39] – a phenotype that is reversed by the administration of an IFN-β blocking antibody or by loss of the type I interferon receptor (IFNAR) [39,40]. C57BL/6 mice infected with *Mtb* produce lower levels of IFN-β following infection, but even in this context, loss of MAVS or IFNAR results in decreased bacterial burden and increased survival of infected animals [25,26]. Thus, activation of antiviral responses within the host seems to actively interfere with an effective antibacterial response to *Mtb*, and suggests that *Mtb* might introduce nucleic acids into the host cell as a virulence strategy.

Ubiquitin signaling plays an important role in orchestrating innate immune responses, and regulates both antibacterial and antiviral responses in *Mtb*-infected macrophages. Ubiquitin ligases such as PARKIN and SMURF are critical in targeting *Mtb* for autophagy [41–44]. Separately, the ubiquitin ligase CBL has been shown to regulate the expression of type I interferon following *Mtb* infection [45], and is targeted by the secreted *Mtb* virulence factor LpqN. LpqN is dispensable for growth in axenic culture, but is required for *Mtb* replication in mice [45]. Proteomic analyses identified a physical interaction between LpqN and CBL, and the growth of the attenuated *lpqN Mtb* mutant was rescued by disruption of host *Cbl*. Thus, CBL acts to maintain the antibacterial capacity of an *Mtb*-infected macrophage, but how it regulates this process is unknown.

In this study we investigated the mechanisms by which CBL regulates immune signaling. We found that it controls macrophage responses to multiple stimuli and represses the expression of numerous antiviral effectors. We then used proteomics to identify potential CBL substrates in macrophages as they respond to *Mtb* infection and identified the endoplasmic reticulum-localized signaling adapter Fas-associated factor 2 (FAF2) as a protein that undergoes CBL-dependent ubiquitylation. Notably, while CBL-deficient macrophages are more permissive for *Mtb* growth, disrupting FAF2 in this context reverses this phenotype and enhances delivery of *Mtb* to the lysosome. Taken together, this suggests that the FAF2 pathway might be hijacked by *Mtb* to disrupt host defenses*,* and that CBL acts to constrain FAF2 to preserve antibacterial functions.

## Results

### CBL broadly suppresses antiviral effector expression

**Fig 1:**
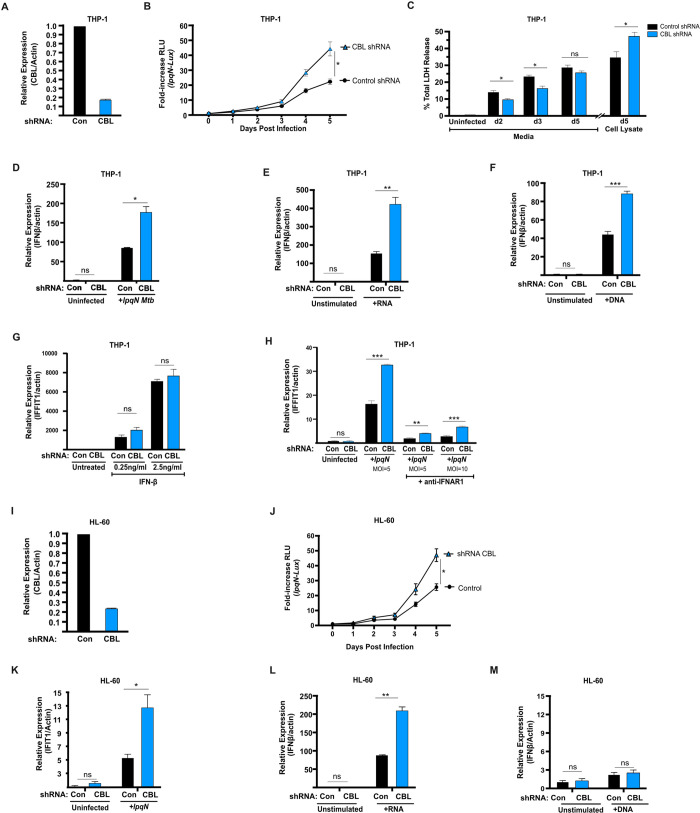
CBL suppresses antiviral responses in human cells. **(A)** CBL expression analyzed by RT-qPCR analysis of THP-1 cells expressing CBL-specific shRNA or a control non-targeting shRNA in cells differentiated for 72 h prior to infection with PMA and VitD. **(B)** Luminescent growth assay of CBL-sensitive *lpqN Mtb* mutant carrying the *LuxBCADE* operon in macrophages differentiated for 72 h prior to infection with PMA and VitD. **(C)** LDH release assay for cell death; the percentage of total LDH activity released at each time is plotted. Remaining viable cells were lysed on day 5 post-infection and LDH activity measured in the lysate. Media from differentiated but uninfected cells after 48 h of culture was analyzed in parallel. **(D-H)** RT-qPCR analysis of differentiated THP-1 cells 6 h after infection with *lpqN Mtb* or exposure to different stimuli as indicated. **(I)** RT-qPCR analysis of CBL expression in HL-60 cells differentiated for 72 h in PMA and VitD. **(J)** Luminescent growth assay of *lpqN Mtb* in HL-60 cells. **(K-M)** RT-qPCR analysis of differentiated HL-60 cells 6 h after exposure to different stimuli as indicated. Error bars denote SEM of technical replicates; statistical significance was evaluated by two-tailed t-test and indicated on plot: *p ≤ 0.05, **p ≤ 0.01, ***p ≤ 0.001, ns p > 0.05. Representative (median) data of three or more independent experiments are shown.

Prior work has shown that CBL suppresses host antiviral responses in mouse primary bone-marrow-derived macrophages (BMDMs) following *Mtb* infection [45]. We began by assessing whether this role of CBL was evolutionarily conserved, with CBL also regulating these processes in human cells. We used shRNA to deplete CBL mRNA in the human THP-1 cell line that differentiates into macrophage-like cells after stimulation with phorbol-12-myristate-13-acetate (PMA) and 1,25-dihydroxy-vitamin D3 (VitD), and found an ~ 85% reduction in CBL mRNA levels relative to control cells expressing a non-targeting shRNA (Fig 1A). We then assessed whether loss of CBL in human macrophages would similarly rescue the growth of the CBL-sensitive *lpqN* mutant *Mtb* strain (CDC1551 background) which lacks the effector that normally antagonizes CBL function, and which is attenuated in both mouse BMDMs and in mice [45]. Following differentiation, we infected THP-1 control cells and CBL-depleted cells. To monitor bacterial replication, we used the *lpqN Mtb* mutant carrying a *LuxBCADE* bioluminescent reporter operon [45], and monitored bacterial growth over time by quantifying luminescence. Consistent with prior observations in mouse BMDMs, we found that loss of CBL also rescued the growth of the *lpqN* mutant in human macrophages (Fig 1B). The increased bacterial replication was confirmed by direct colony forming unit (CFU) enumeration (S1A Fig), and independently verified using a second independent shRNA in THP-1 cells (S1B Fig). In addition, we monitored cell death through the release of lactate dehydrogenase (LDH) into the culture media, and saw no defects in cell viability of CBL-deficient cells following infection with the *lpqN* mutant *Mtb* (Fig 1C).

We next determined whether expression of antiviral effectors was also regulated by CBL in human cells. Consistent with prior observations in mouse macrophages, we found that depletion of CBL resulted in an increased expression of IFN-β in THP-1 cells following infection with the *lpqN Mtb* mutant (Fig 1D). We next used defined PAMPs to selectively stimulate distinct pathways leading to IFN-β activation to determine whether CBL was regulating a single specific sensor pathway or whether it regulated multiple pathways. Work from several groups has shown that although *Mtb* activates numerous host sensors, the DNA sensor cGAS is required for *Mtb*-induction of IFN-β expression, with the RNA sensor RIG-I contributing to sustained activation [24,25,31,32]. To assess whether CBL is regulating either DNA or RNA sensing pathways, we separately transfected THP-1 macrophages with either dsDNA to activate cGAS, or with 5′-phosphorylated RNA to activate RIG-I. We found that in response to either cytoplasmic DNA or to 5’ phosphorylated RNA, loss of CBL resulted in an increased expression of IFN-β (Fig 1E and 1F). We also tested whether CBL regulates host cell responsiveness to IFN-β itself. We stimulated cells with varying doses of IFN-β and monitored expression of IFIT1, a canonical ISG. Unlike *Mtb* infection, DNA stimulation, and RNA stimulation, we found that, following isolated IFN-β stimulation, CBL had no significant effect on gene expression (Fig 1G). We also tested whether CBL still regulated downstream genes when IFN-β signaling was disrupted in *Mtb*-infected macrophages. We treated cells with an antibody that blocks IFNAR1, a component of the type I interferon receptor, infected cells with *lpqN* mutant *Mtb* that cannot antagonize CBL, and monitored expression of IFIT1. We observed robust activation of IFIT1 in infected cells, with elevated levels in CBL-deficient cells. In the presence of blocking antibody, we found the expected decrease in IFIT1 expression. However, in this context we still observe a CBL-regulated component of IFIT1 expression, with elevated IFIT1 levels in the CBL-deficient cells. This suggests that there exist at least two regulatory pathways, with some changes secondary to elevated IFN-β expression in CBL-deficient cells, and other changes that are independent of type I interferon.

To confirm the role of CBL in human cells, we also evaluated CBL in a second human macrophage cell line, HL-60. Using shRNA to deplete CBL in HL-60 cells (Fig 1I) we found that loss of CBL similarly rescued the growth of the *lpqN* mutant (Fig 1J). In HL-60 cells we also observed a role for CBL in regulating IFN-β expression following infection, and following RNA stimulation (Fig 1K and 1L). However, following DNA stimulation, HL-60 cells only weakly induced IFN-β, and did so independently of CBL (Fig 1M). Taken together, these data support a model where the role of CBL in innate immunity is evolutionarily conserved, and it acts to suppress expression of antiviral effectors such as IFN-β in multiple physiologic conditions.

**Fig 2:**
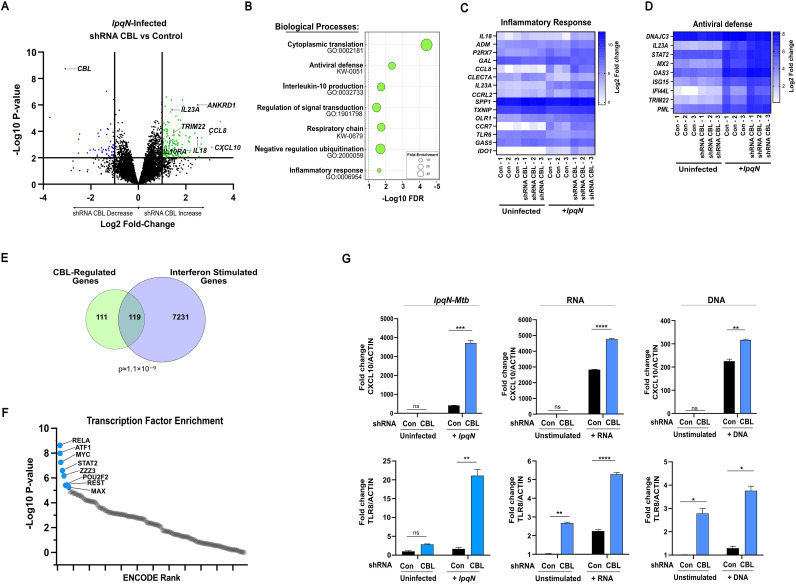
CBL regulates a complex transcriptional program. **(A)** RNA-Seq gene expression analysis of THP-1 macrophages expressing CBL-specific shRNA vs control non-targeting shRNA 6 h after infection with *lpqN* mutant *Mtb*. **(B)** Biological processes transcriptionally regulated by CBL. **(C,D)** Differential expression of immunity-related genes in CBL-deficient cells. **(E)** Comparison of CBL-regulated genes with known ISGs [46]. **(F)** ENCODE Transcription factor binding site enrichment of CBL-regulated genes. **(G)** RT-qPCR analysis of CBL-regulated genes after indicated stimuli. Differentially-expressed genes for analyses in (B-E) defined by RNA-Seq FDR < 0.05 and log_2_ fold-change>0.5. RNA-Seq analysis was performed on 3 independent experiments. For RT-qPCR, error bars denote SEM of technical replicates; statistical significance assessed with two-tailed t-test and indicated on plot. Representative (median) data of three independent experiments are shown. *p ≤ 0.05, **p ≤ 0.01, ***p ≤ 0.001, ns p > 0.05.

We next sought to determine whether CBL selectively regulated a restricted group of genes, such as IFIT1 and IFN-β, or whether it orchestrated broad transcriptional changes in macrophages as they responded to *Mtb*. To assess this, we infected THP-1 cells with *Mtb*, using the *lpqN* mutant strain so that CBL activity would be unconstrained by the bacterial effector. We infected both control and CBL-deficient THP-1 cells for 6 h, a time at which many immune-related genes are strongly induced, and prepared RNA for expression profiling by RNA-Seq. As expected, infection of control cells triggered widespread gene expression changes with upregulation of 735 genes and downregulation of 423 genes (FDR ≤ 0.05, log_2_ fold-change>0.5) and caused strong induction of both antibacterial and antiviral effectors (S1C Fig and S1 Table). CBL-deficient cells regulated most of these genes in a similar manner following infection with the *lpqN Mtb* mutant that cannot antagonize CBL. However, a set of genes showed distinct CBL-dependent transcriptional changes, with 204 genes hyperactivated and 62 genes depressed in CBL-deficient cells following infection (Fig 2A and S1 Table).

We further analyzed the set of genes regulated by CBL to evaluate which cellular processes were being affected, and what transcription factors might be mediating the regulatory effects of CBL. We performed systematic pathway enrichment analysis using the Gene Ontology and KEGG Databases and identified several clusters of functionally-related genes regulated by CBL. Supporting the hypothesis that CBL broadly suppresses antiviral responses, we found a significant over-representation of antiviral defense genes in the set of CBL-regulated genes (10-fold over-representation, FDR < 0.02). We also found significant associations with several other pathways including IL-10 production and global protein translation (Fig 2B–2D). In addition, we analyzed the set of genes transcriptionally regulated by CBL to determine whether any associations existed between these genes and characterized transcriptional regulators. We assessed this by systematically querying the ENCODE Chip-Seq database [47] with the set of genes regulated by CBL. In uninfected cells, there were no transcriptional regulators enriched at these genes. In contrast, in cells infected with the *lpqN Mtb* mutant, analysis of the 266 CBL-responsive genes using ENCODE showed an association with several transcriptional regulators (Fig 2F). The most significant association was seen for the NF-κB family member RELA, with an odds-ratio (OR) of 2.6 (p ≤ 1E-9) found at a set of immune-related genes, with additional associations noted for STAT2 (OR=5.3, p ≤ 2E-7), ATF1 (OR=2.5, p ≤ 1.1E-8), and MYC (OR=2.1, p ≤ 5E-8), suggesting that these transcription factors might be mediating the transcriptional effects of CBL.

We also assessed whether genes we identified as regulated by CBL during *Mtb* infection were also regulated by CBL in response to other stimuli. We selected TLR8 and CXCL10, two genes identified by our RNA-Seq analysis whose expression was suppressed by CBL, and examined their regulation in cells exposed to other PAMPs - either cytoplasmic dsDNA or 5’-phosphorylated RNA. In both cases we found hyperactivation of these genes in CBL-deficient cells, although the patterns differed. For TLR8 we detected basal de-repression in unstimulated cells and hyperactivation following either infection or nucleic acid stimulation. For CXCL10, there was no basal de-repression, and while CXCL10 was strongly regulated by CBL during infection, it showed only modest increases in expression in CBL-deficient cells stimulated with DNA or RNA (Fig 2G). Taken together, these results demonstrate that CBL regulates a broad set of antiviral response genes in macrophages, along with regulation of several other processes, and that it does so in response to multiple stimuli.

### CBL enzymatic activity is required to regulate macrophage responses to *Mtb*

We next sought to determine the mechanisms by which CBL regulates immune responses. CBL is a RING-domain E3 ligase that was initially characterized as a proto-oncogene, ubiquitylating activated receptor tyrosine kinases to target them for degradation [48–52]. Subsequent studies using an enzymatically-inactive CBL point mutant showed that CBL also has important non-enzymatic functions, and can act as a signaling scaffold that potentiates phosphatidylinositol 3-kinase and SRC-family kinase activity [53]. This non-catalytic function plays an important role in vivo, as several of the developmental defects in *Cbl*^*-/-*^ mice could be rescued by expression of catalytically-inactive CBL [54].

**Fig 3:**
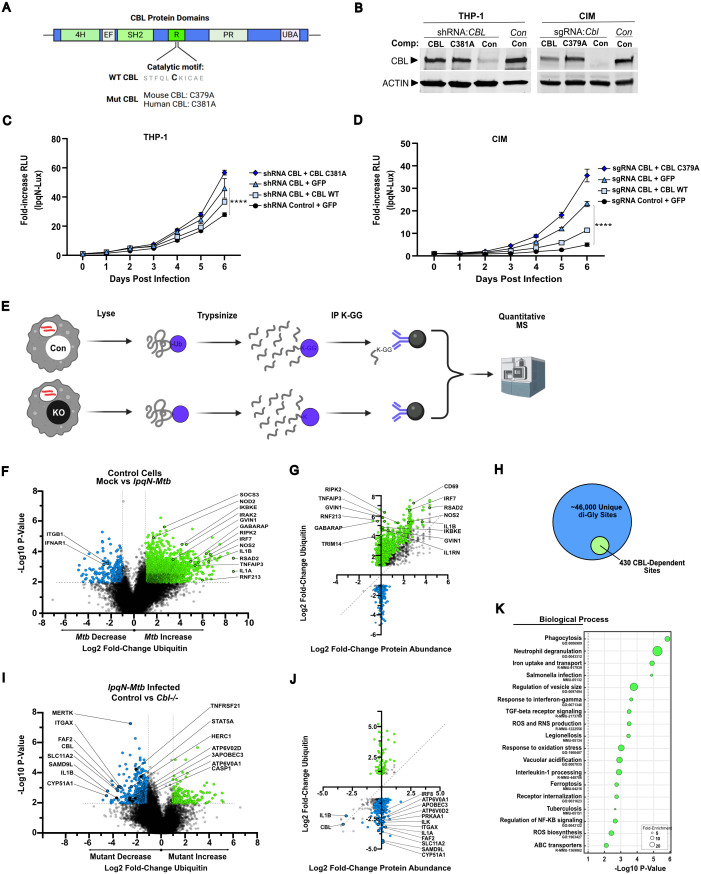
CBL-dependent ubiquitylation is required to restrict growth of the CBL-sensitive *lpqN* mutant *Mtb* in mouse and human macrophages. **(A)** CBL domain structure and catalytic site mutants. **(B)** Immunoblot of wild-type and mutant CBL isoforms expressed from lentiviral vectors in CBL-deficient human (THP-1) and mouse (CIM) macrophages. **(C, D)** Luminescent growth assay of *lpqN Mtb* in CBL-deficient macrophages in which CBL expression was restored with either wild-type or mutant CBL. **(E)** Experimental design for ubiquitylation proteomics using di-Gly MS. Following trypsinization, ubiquitin-modified peptides contain a fragment of ubiquitin (di-Gly) that remains conjugated to the ubiquitylated Lys residue. Di-Gly-containing peptides are then immunoprecipitated and quantified by MS. (F) di-Gly MS analysis of ubiquitylation in control CIM cells expressing a non-targeting sgRNA 6 h post-infection with the *lpqN Mtb* mutant. Green (increased) and blue (decreased) indicate peptides with p-value ≤0.01 and log_2_ fold-change >2. **(G)** Comparison of ubiquitylation versus total-protein changes in control cells following infection. Green (increased) and blue (decreased) denote data points where the change in di-Gly peptide abundance was > 3 standard-deviations greater than the change in protein abundance. **(H)** Subset of CBL-dependent changes. (I) di-Gly MS analysis of ubiquitylation 6 h post-infection in control vs. *Cbl*^-/-^ cells infected with *lpqN Mtb*. **(J)** Ubiquitylation versus total-protein changes in control vs. *Cbl*^-/-^ cells infected with *lpqN Mtb.* **(K)** Biological process of proteins with CBL-dependent ubiquitylation. MOI = 0.8 for growth assays, and MOI = 10 for di-Gly MS. Di-gly MS was used to analyze 3 independent experiments. For growth assays, representative (median) data of three or more independent experiments are shown; p-value determined by two-tailed t-test and error bars represent SEM of technical replicates. *p ≤ 0.05, **p ≤ 0.01, ***p ≤ 0.001, ns p > 0.05. Schematic images were prepared with Biorender.

To test whether CBL was acting enzymatically to ubiquitylate key substrates during innate immune responses, or whether it was acting as a signaling adapter protein, we performed genetic complementation analysis in CBL-deficient macrophages, re-introducing either wild-type CBL or catalytically inactive mutants (Fig 3A). To study human macrophages, we used shRNA-expressing CBL-deficient THP-1 cells. To study mouse macrophages we used Cas9-expressing conditionally-immortalized macrophages (CIMs) which are mouse myeloid precursor cells that carry a HOXB8-ER estrogen-regulatable transcription factor. This maintains the cells as immortalized precursors in the presence of estrogen, but upon estrogen withdrawal allows differentiation into macrophages that closely recapitulate the physiology of primary mouse BMDMs [55]. These cells display high-efficiency genome editing, and transduction of CIM cells with a CBL-specific sgRNA led to a > 95% reduction in CBL protein levels in the population (Fig 3B).

For both mouse and human cells, we used lentiviral vectors to deliver either catalytically inactive CBL (human C381A, mouse C379A) or WT CBL [56–60]. In both cases, CBL expression was restored to ~50% of endogenous levels, without evidence of destabilization of the mutant protein (Fig 3B). As seen previously, growth of the attenuated *lpqN* mutant that is unable to antagonize CBL was restored in CBL-deficient macrophages [45]. In both mouse and human macrophages, re-introduction of wild-type CBL significantly restricted the growth of the *lpqN* mutant *Mtb* (Fig 3C and 3D). In contrast, re-introduction of catalytically-inactive CBL failed to complement the defective host response, demonstrating that enzymatic activity is needed for CBL function in this context. We also noted that CBL-deficient cells reconstituted with inactive CBL had higher bacterial loads than CBL-deficient cells, suggesting that catalytically-inactive CBL might act as a dominant-negative isoform.

### Identifying potential CBL substrates

Given that the enzymatic activity of CBL was necessary to regulate immune signaling, we next sought to identify CBL substrates in this context. Several growth factor receptors including EGFR, and CSF1R have been characterized as CBL substrates in other settings [48–50], however, the CBL substrates during innate immune responses are not known. We used *Cbl*^*-/-*^ CIM cells to study ubiquitylation because of the near-complete loss of CBL protein in the polyclonal population of cells expressing a CBL-specific sgRNA, and their greater defect in restricting growth of the *lpqN* mutant *Mtb,* relative to human cells partially depleted of CBL via shRNA (Fig 3B–3D). We infected cells for 6 h, and added the proteasome inhibitor bortezomib for the final 2 h prior to sample collection to stabilize ubiquitylated proteins and facilitate their detection.

We then used di-Gly enrichment and quantitative mass spectrometry (di-Gly MS) to globally monitor changes in ubiquitylation in *lpqN* mutant *Mtb*-infected cells (Fig 3E). Lysates were prepared from uninfected and infected cells, and from control and *Cbl*^*-/-*^ cells. We then digested the lysates with trypsin and enriched ubiquitylated peptides by immunoprecipitation with an antibody that recognizes the di-Gly remnant of ubiquitin that remains conjugated to the ubiquitin-modified Lys residue (Fig 3E), and analyzed the samples by liquid chromatography tandem MS (LC-MS3), using label-free quantification of the MS3 spectra [61–66]. Of note, the ubiquitin-like proteins ISG15 and NEDD8 leave an indistinguishable di-Gly Lys remnant. However, these other modifications account for only ~5% of identified di-Gly modified residues, and the vast majority of di-Gly sites are ubiquitin-modified [67–69]. In addition, since CBL is a ubiquitin-specific ligase, any CBL-dependent di-Gly modifications should represent CBL-dependent ubiquitylation. Thus, we will hereafter refer to di-Gly-modified sites as ubiquitylation, recognizing that a small percentage of sites modified by ligases other than CBL are modified by ISG15 or NEDD8.

In control cells, our analysis provided a detailed description of the dynamic ubiquitylation changes that occur during *Mtb* infection. Overall, we identified ~76,000 unique peptides in macrophages, encompassing ~46,000 unique di-Gly-modified sites. In control cells, we saw numerous ubiquitin changes*,* with a total of 1,793 ubiquitylation sites increasing (log_2_ fold-change >1, FDR < 0.05) and 197 sites decreasing 6 h after infection (Fig 3F and S2 Table). Since the quantity of a ubiquitylated peptide in a sample can change because of either a change in the stoichiometry of ubiquitylation at a given site, or because of a change in abundance of the protein itself, we sought to deconvolute these processes. We examined changes in protein abundance in the same samples by quantitative MS (S3 Table) and analyzed the change in ubiquitylation at each site relative to the change in protein expression. This analysis demonstrated a range of stoichiometry changes. For some proteins, such as RSAD2 and IL1B, the increased quantity of ubiquitylated peptides was largely due to increased protein expression (Fig 3G). Conversely, proteins like IKBKE and GABARAP had large changes in ubiquitylation stoichiometry with minimal change in overall protein levels. Overall, changes in abundance of a ubiquitylated peptide and protein abundance were poorly correlated (R^2^ = 0.36) with changes in peptide abundance being driven by changes in the stoichiometry of ubiquitylation in roughly 70% of cases.

These analyses probe deeper into the ubiquitin-modified proteome of macrophages than prior studies, with roughly 10-fold more sites identified, capturing numerous dynamic ubiquitin signaling events as macrophages respond to a virulent pathogen [70]. These data provide important new insights. Our analyses revealed a number of well-established immune regulators with previously unknown ubiquitin modifications. This includes the endosomal peptidoglycan transporter SLC15A3 and cytoplasmic peptidoglycan sensor NOD2. It also includes a surprising number of secreted cytokines including TNF, TNFSF13B, CCL2 and CXCL2. In addition, these analyses have pinpointed the ubiquitinated residues of other regulators such as CASP1 and IL1A where prior immunoblot studies demonstrated ubiquitin modifications but the exact ubiquitylation sites have proven elusive (S2 Table). Taken together, these observations suggest the hypothesis that ubiquitylation may regulate some of these processes, including previously unexpected immune signaling processes such as the stability or secretion of key cytokines.

We next examined ubiquitylation in CBL-deficient macrophages. As expected, the loss of CBL did not change the abundance of most ubiquitylated peptides - consistent with a complex ubiquitin signaling landscape involving numerous E3 ligases. However, there was also a clear subset of peptides that underwent CBL-dependent ubiquitylation, including well-established CBL substrates such as the receptors MET and CSF1R. As anticipated, most changes seen in *Cbl*^-/-^ cells were peptides with decreased ubiquitylation; there were 430 ubiquitylation sites that decreased (log_2_ fold change > 1.0, FDR < 0.05) and 90 sites that increased in abundance (Fig 3H and 3I). As was seen in control cells, the majority of ubiquitylation changes were due to changes in the stoichiometry of ubiquitylation, with very little correlation between changes in abundance for ubiquitylated peptides and protein expression (R^2^ = 0.09, Fig 3J).

Systematic analysis of the proteins with CBL-dependent ubiquitylation demonstrated the enrichment of several pathways and processes; some had clear immune-related functions such as regulation of phagocytosis (7.1-fold over-representation, p ≤ 0.01), vacuolar acidification (13.7-fold, p = 0.02), NF-κB regulation (7.2-fold, p = 0.02), and IFNGR signaling (4.2-fold, p ≤ 0.01, Fig 3K). There were also factors known to be involved in the immune response to *Legionella pneumophila* (5.3-fold, p ≤ 0.01), *Salmonella enterica* (3.2-fold, p ≤ 0.001), and *Mtb* itself (3.1-fold, p < 0.01). Several other unanticipated pathways were also over-represented in the set of proteins with CBL-dependent ubiquitylation, including endoplasmic reticulum-associated protein degradation (ERAD) (6.2-fold over-representation, p ≤ 0.001) and iron uptake (6.1-fold, p ≤ 0.001). Taken together, these findings suggest that the set of proteins ubiquitylated by CBL extends far beyond its established role in growth-factor signaling at the plasma membrane and includes cellular processes throughout the cytoplasm and on membrane-bound organelles.

### Function of proteins with CBL-dependent ubiquitylation

**Fig 4:**
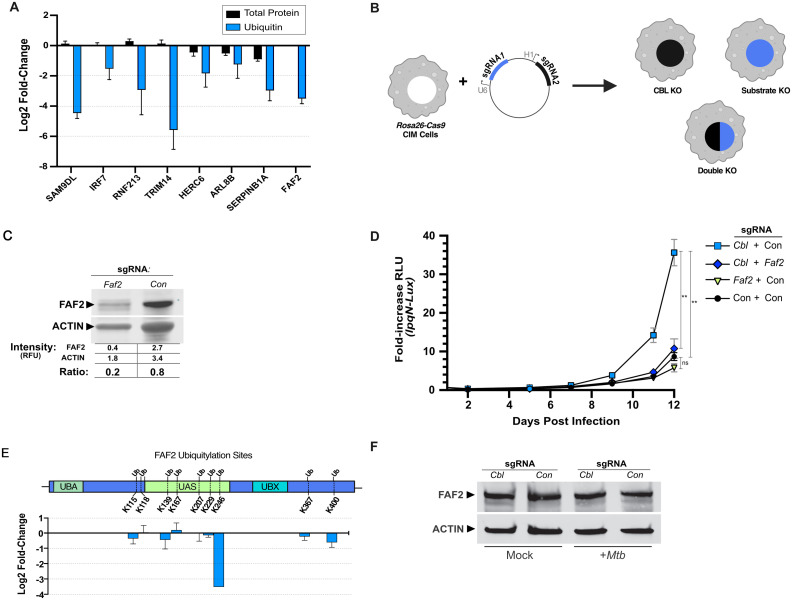
FAF2 promotes *Mtb* replication in the absence of CBL. **(A)** Examples of changes in ubiquitylation and total protein for immune regulators in *Cbl*^*-/-*^ CIM cells infected with the *lpqN Mtb* mutant that is unable to antagonize CBL at 6 h post-infection. In the case of proteins with multiple ubiquitylated peptides, the peptide with greatest statistically-significant fold-change is shown. **(B)** Experimental design for genetic epistasis analysis between *Cbl* and its putative substrates. Cas9-expressing CIM cells were transduced with combinations of sgRNA targeting CBL, sgRNA targeting potential substrates, or control non-targeting sgRNA to create single- and double-mutant cells for genetic epistasis analysis. **(C)** Immunoblot of FAF2 protein in CIM cells expressing either non-targeting or *Faf2*-specific sgRNA. **(D)** Luminescent growth assay of CBL-sensitive *lpqN Mtb*. **(E)** Location of ubiquitylated residues on FAF2 and log_2_ fold-change of ubiquitylation at each residue in *Mtb*-infected *Cbl*^-/-^ cells vs. control cells. **(F)** Immunoblot of FAF2 protein levels in *lpqN* mutant *Mtb*-infected control and *Cbl*^-/-^ cells. Error bars denote SEM of technical replicates; statistical significance was evaluated by two-tailed t-test. Representative (median) data of 4 independent experiments is shown. *p ≤ 0.05, **p ≤ 0.01, ***p ≤ 0.001, ns p > 0.05.

We next sought to identify which proteins with CBL-dependent ubiquitylation played important roles during *Mtb* infection. We identified a subset of 43 proteins that had peptides with at least an 80% reduction in ubiquitylation in *Cbl*^*-/-*^ cells. Of these, 20 had annotated functions related to innate immunity, and we began to analyze this set of proteins for CBL-dependent functions during *Mtb* infection. We hypothesized that if a protein is an important substrate of CBL, then loss of that protein should either phenocopy the CBL mutant (if positively regulated by CBL) or reverse the CBL phenotype (if inhibited by CBL). To analyze each of these factors we created a lentiviral vector with two sgRNA cassettes, thereby allowing us to generate mutant CIM cell lines lacking either CBL or a putative CBL substrate, as well as double-knockout (DKO) cells lacking both simultaneously (Fig 4A and 4B). Editing efficiency was determined by either TIDE analysis of genomic DNA [71] or by immunoblot of the targeted protein (Fig 4C). Genome editing at the *Cbl* locus remained highly-efficient in DKO macrophages. For the initial set of DKO cell lines analyzed, 12 of 15 loci encoding putative CBL substrates had genome editing efficiencies of >70% and were analyzed for their ability to reverse the *Cbl*^*-/-*^ phenotype. We then infected each of these mutant cell lines with the CBL-sensitive *lpqN Mtb* mutant and assessed bacterial growth. As expected, bacterial growth was more rapid in CBL-deficient cells (Fig 4D), and in most cases, loss of a putative substrate in these CBL-deficient cells had little to no effect on bacterial replication (S2 Fig).

However, disruption of the Fas-associated factor 2 (*Faf2*) locus in CBL-deficient cells significantly restored the antibacterial capacity of these cells, enabling the *Cbl*^*-/-*^*; Faf2*^*-/-*^ DKO cells to restrict the growth of the CBL-sensitive *lpqN* mutant *Mtb*. FAF2 is an ER-localized signaling adapter protein, and our di-Gly MS analysis found it to be heavily ubiquitylated in macrophages, with 9 different ubiquitylated Lys residues. In *Cbl*^*-/-*^ cells, a single residue (K246) showed a specific loss of ubiquitylation, with other Lys residues relatively unaffected (Fig 4E). In contrast to the other genes tested, disruption of FAF2 in CBL-deficient cells restored the ability of these cells to restrict replication of the *lpqN* mutant, with bacterial growth rates approaching that seen in control macrophages (Fig 4D). Given the established role of CBL in targeting growth factor receptors for degradation, we tested the idea that CBL similarly targets FAF2 for degradation through ubiquitylation. However, FAF2 protein levels did not change following infection and were unaltered in *Cbl*^*-/-*^ macrophages (Fig 4F), suggesting that CBL-dependent ubiquitylation modulates FAF2 activity rather than triggering its degradation.

We also analyzed the growth of wild-type *Mtb* in *Faf2*^*-/-*^ single mutant cells, *Cbl*^*-/-*^ single mutant cells, and DKO macrophages. We had previously found for *Mtb* strains expressing *lpqN* (either wild-type *Mtb* or the *lpqN* complemented with a wild-type copy of *lpqN*) that host CBL had only minimal effects on bacterial growth [45]. Here we find that this small increase in the ability of wild-type *Mtb* to replicate in *Cbl*^*-/-*^ cells is decreased by loss of *Faf2* in DKO cells (S3A Fig). Interestingly, in *Faf2*^*-/-*^ single-mutant cells we also detected a small decrease in *Mtb* replication, further supporting the idea that FAF2 promotes *Mtb* replication. Taken together, these genetic analyses show that while multiple proteins undergo CBL-dependent ubiquitylation, FAF2 seems to be an important regulatory hub downstream of CBL as macrophages respond to *Mtb* infection.

**Fig 5:**
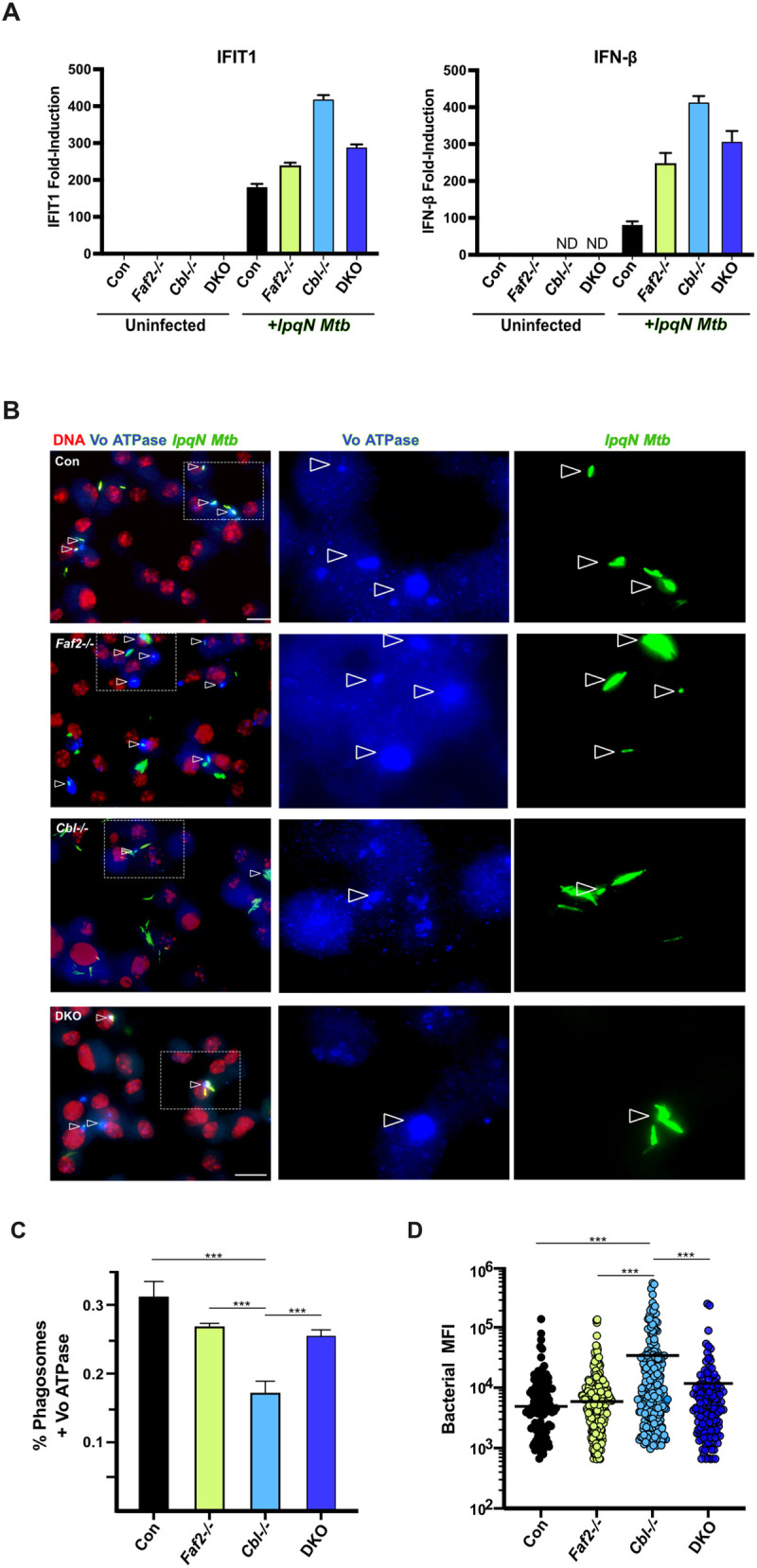
FAF2 impairs phagosome trafficking. **(A)** RT-qPCR analysis of IFIT1 and IFN-β expression in *Faf2*^*-/-*^, *Cbl*^*-/-*^ and DKO macrophages 6 h after infection by *lpqN* mutant *Mtb*. **(B)** Microscopy analysis of macrophages infected with *lpqN Mtb* (MOI = 1) using Immunofluorescence to detect ATP6E, a subunit of the vacuolar ATPase complex (Vo ATPase)*.* Arrowheads indicate *Mtb*-Vo ATPase colocalization events; scale bar = 20 µm. **(C)** Quantification of Vo ATPase colocalization. **(D)** *Mtb* fluorescent intensity of individual phagosomes. For microscopy >400 phagosomes were quantified for each condition. Error bars denote SEM of technical replicates; statistical significance was evaluated by two-tailed t-test in (A) and (C), and by non-parametric Wilcoxon signed-rank test in (D); *p ≤ 0.05, **p ≤ 0.01, ***p ≤ 0.001, ns p > 0.05. Each of these experiments was repeated twice.

We next sought to determine the mechanism by which FAF2 regulates antibacterial responses to *Mtb.* We first tested the hypothesis that FAF2 mediates the elevated expression of antiviral effectors in CBL-deficient macrophages, and that this was responsible for the impaired antibacterial responses. We analyzed expression of IFIT1 and IFN-β in *Faf2*^*-/-*^, *Cbl*^*-/-*^ and DKO macrophages infected with *lpqN Mtb,* and found, as expected, a significant increase in expression of both genes in *Cbl*^*-/-*^ cells. However, expression of these antiviral effectors did not correlate with bacterial restriction, as expression was only slightly diminished in DKO cells, and in the case of IFN-β, was actually elevated in *Faf2*^*-/-*^ single-mutant cells (Fig 5A).

Thus, we next evaluated whether FAF2 might control other processes, and tested whether phagosome maturation was regulated by FAF2. We infected control macrophages, macrophages singly deficient in either *Cbl*^*-/-*^ or *Faf2*^*-/-*^ alone, or DKO macrophages with the *lpqN Mtb* mutant that is unable to antagonize CBL, and used immunofluorescent microscopy to measure colocalization between *Mtb*-containing phagosomes and the lysosomal marker ATP6E, a subunit of the vacuolar ATPase complex (Vo ATPase). In *Cbl*^*-/-*^ cells we saw a decreased fraction of *Mtb*-containing phagosomes co-localized with the Vo ATPase relative to control macrophages, correlating with the greater replication of the *lpqN Mtb* mutant in these cells. (Fig 5B and 5C). We also noted that phagosomes in *Cbl*^*-/-*^ macrophages contained larger numbers of bacteria as measured by the mean fluorescent intensity (MFI) of the bacteria in individual phagosomes (Fig 5D). In DKO macrophages, where bacterial growth restriction is restored, Vo ATPase accumulation on *Mtb* phagosomes was also restored (Fig 5C), demonstrating that FAF2 is able to modulate phagosome-lysosome fusion. We confirmed these changes in lysosome colocalization in independent experiments with an acidotropic lysosome stain (S3B and S3C Fig) with similar results. Taken together, these data suggest a model where CBL normally constrains FAF2 activity in *Mtb*-infected cells, thus promoting phagosome maturation. However, if CBL is absent, then unrestrained FAF2 activity impairs phagosome maturation, with a consequent increase in bacterial replication.

## Discussion

### Antiviral versus antibacterial cellular responses

How an immune cell determines the type of pathogen it is encountering, and translates that into an appropriate response, is an unresolved question in immunology. *Mtb* simultaneously exposes an infected macrophage to an array of PAMPs. Some are distinctively bacterial molecules such as peptidoglycan or lipoarabinomannan, which activate NOD2 and TLR2 respectively, and result in the release of cytokines with potent antibacterial activities such as TNF and IL1B [6–9,11,12]. However, *Mtb*, as well as a number of other intracellular bacterial pathogens, including *Listeria monocytogenes* and *Chlamydia trachomatis,* also activate cytosolic nucleic-acid sensors and induce the expression of type I interferon and antiviral effectors [72–75]. While type I interferon potently inhibits viruses and can inhibit the growth of some bacteria such as *C. trachomatis*, it impairs antibacterial responses to *Mtb* and *L. monocytogenes* in macrophages and in mice [72–75]*.*

The observation that antiviral responses can actively antagonize host antibacterial capacity suggests pathogens infecting macrophages like *Mtb* and *L. monocytogenes* might seek to subvert this process as a virulence strategy. In the case of *Mtb*, it seems to do so through several mechanisms. First, upon perforating its phagosome, *Mtb* exposes bacterial DNA to the host cell cytoplasm which results in IRF3 activation of hundreds of downstream genes, including IFN-β [24–31]. Second, in parallel, *Mtb* also releases bacterial RNA into the host cytoplasm, thereby activating a similar RIG-I-dependent activation of IRF3 and IRF7, and antiviral effector expression [25]. *Mtb* also introduces the LpqN virulence factor into host cells which interferes with the ability of the CBL ubiquitin ligase to regulate this process, thereby amplifying antiviral responses at the expense of an effective antibacterial response [45].

Exactly how the expression of antiviral effectors antagonizes the antibacterial state of a macrophage is unclear. Integrating the results of different studies that examined different viral response pathways suggests that the transcriptional program is likely to be comprised of several distinct modules, as the perturbation of different regulators causes distinct *Mtb*-related phenotypes. For example, in isolated ex vivo macrophages, disruption of CBL, TRIM14, or IRF7 alters the expression of antiviral effectors and alters the ability of a macrophage to restrict *Mtb* replication [25,45,76,77]. In contrast, similar disruption of RIG-I, SP140 or IFNAR1 in isolated ex vivo macrophages has no effect on *Mtb* replication - but profoundly alters *Mtb* susceptibility in mice [77–80]. Thus, although all of these pathways activate IFN-β, their distinct *Mtb*-related phenotypes suggest that they also have divergent effects on other host processes that drive distinct physiologic changes in *Mtb*-infected macrophages. The strong phenotypes seen in vivo, that are absent in some ex vivo experiments, also suggests that during the complex multicellular immune response in vivo there exists a distinct environment where type I interferon signaling becomes a dominant factor. While it is possible that some of these differences could be due to methodologic variability between laboratories, in several cases the strong effects of one antiviral regulator and no effect of other regulators has been seen within individual studies [40,45], suggesting important underlying biological differences.

### FAF2 function

Although the mechanisms by which CBL regulates growth factor signaling are known in detail, the mechanisms by which it regulates the innate immune response are not. We found that the enzymatic activity of CBL was needed for its ability to regulate immune responses and restrict the growth of the *lpqN* mutant. We also identified the signaling adapter protein FAF2 as a critical intermediary factor, as disruption of FAF2 rescued antibacterial responses in CBL-deficient cells, increasing phagosome maturation. We do note that expression of IFN-β was not decreased by FAF2, indicating that there are at least two pathways regulated by CBL.

FAF2 is known to regulate a diverse set of physiologic processes. It was originally characterized as a member of a family of ubiquitin regulatory X (UBX) domain proteins regulating the endoplasmic-reticulum-associated degradation pathway (ERAD) [81], but was also subsequently shown to act as a positive regulator of STING1, MTOR, and NF-κB [82–84]. Our data suggest a scenario where FAF2 activity might somehow be hijacked by *Mtb* to impair host defenses through one of these pathways, and that CBL normally acts to constrain FAF2.

It is also unclear what function the CBL-dependent ubiquitylation of FAF2 at K246 plays, and what ubiquitin linkage type is involved. A straightforward model would have been that CBL inhibits FAF2 by conjugating K48-linked ubiquitin to target FAF2 for proteasomal degradation. However, we find no evidence that FAF2 is degraded. CBL is able to mediate both K48 and K63 linkages, and thus our data suggest that K246 might undergo K63-linked ubiquitylation that results in altered protein-protein interactions with activators of STING1, MTOR, or NF-κB. Future experiments analyzing FAF2 alleles with ubiquitin-site mutations should provide important insight into both the linkage-type and function of FAF2 ubiquitylation.

### Limitations

While our RNA-Seq data show clear CBL-dependent transcriptional changes in CBL-depleted THP-1 macrophages, the response of cell lines such as THP-1 cells to a range of stimuli is often muted relative to primary macrophages [55]. In addition, because of relatively inefficient genome editing in human macrophage cell lines (THP-1, HL-60, U937) we were unable to generate *FAF2*^*-/-*^; CBL^-/-^ double-mutants in human cells to analyze FAF2 in this context. Our MS studies also had some limitations. They identified a large number of peptides with the di-Gly remnant, and while ~95% of di-Gly modifications result from ubiquitylation, NEDD8 and ISG15 both leave identical di-Gly remnants. Thus, our current data are unable to distinguish the small subset of Lys residues modified by other ubiquitin-related proteins. In addition, the linkage-type of the ubiquitin chain conjugated at a particular site also cannot be determined by di-Gly-based proteomics. Finally, for those proteins with CBL-dependent ubiquitylation, we cannot distinguish at this time between direct ubiquitylation by CBL versus indirect effects mediated by another E3 ligase or deubiquitinase that CBL regulates, an analysis that will require complex in vitro ubiquitylation systems with MS analysis of reaction products, which is an important future direction.

## Materials & methods

### KEY REAGENT TABLE

**Table:** 

REAGENT OR RESOURCE	SOURCE	IDENTIFIER
**Antibodies**		
Rabbit c-CBL Antibody	Cell Signaling Technology	Cat # 2747
β-Actin (C4) Alexa Fluor 680	Santa Cruz Biotechnology	Cat # sc-47778 AF680
PTMScan Ubiquitin Remnant Motif (K-ε-GG)	Cell Signaling Technology	Cat # 5562S
FAF2 antibody	Proteintech	Cat # 16251–1-AP
IRDye 800CW Donkey anti-Mouse IgG Secondary Antibody	LI-COR	Cat # 926–32212
IRDye 800CW Goat anti-Rabbit IgG Secondary Antibody	LI-COR	Cat # 926–32211
ATP6E Antibody	Santa Cruz Biotech	sc-20946
Human IFNAR1 Antibody	Antibodies.com	A318850-100
**Bacterial strains**		
CDC1551 *lpqN*:Tn himar1 mutant	BEI Resources	Cat # NR-18454
CDC1551 wild type	Gift from Jeffery Cox, UC Berkeley	
**Mammalian Cell lines**		
THP-1	ATCC	TIB-202
HL-60	ATCC	CCL-240
*HoxB8-ER; Rosa26 Cas9* CIM	Gift from Greg Barton, UC Berkeley	N/A
B16	ATCC	CRL-6475
3T3-MCSF	Gift from Russell Vance, UC Berkeley	
**Chemicals**		
Middlebrook OADC	Thermo Fisher Scientific	Cat # b12351
BD DIFCO Middlebrook 7H9	Becton Dickinson (BD)	Cat # 271310
BD DIFCO Middlebrook 7H10	Becton Dickinson (BD)	Cat # 262710
PTMScan Lys-C Protease	Cell Signaling Technology	Cat # 39003
Sequencing Grade Modified Trypsin	Promega	Cat # V5111
Bortezomib	Cell Signaling Technology	Cat # 2204S
cOmplete, Mini, EDTA-free Protease Inhibitor Cocktail	Sigma Aldrich	Cat # 11836170001
Bovine growth serum	HyClone	SH30541.03
Fetal bovine serum	Corning	5-015-CV
Phorbol 12-myristate 13-acetate (PMA)	Sigma Aldrich	Cat # P8139
1α,25-Dihydroxyvitamin D3 (Vitamin D)	Santa Cruz	Cat # 32222-06-3
SYBR Gold Nucleic Acid Gel Stain	Invitrogen	Cat # S11494
Poly(ethylene glycol) 8000	Sigma	Cat # 81268
Lipofectamine 2000	Invitrogen	Cat # 11668030
Rhodamine B	Sigma	83689-1G
Triton X-100	Sigma	T9284-100ML
Maxima H Minus Reverse Transcriptase	Thermo Scientific	Cat # EP0753
CellTrace Far Red	Thermo Scientific	Cat # C34572
CytoFix Red Lysosomal Stain	AAT Bioquest	Cat # 76485–168
**Consumables**		
500mg Sep-Pak C18 columns	Waters	Cat # WAT036790
PureLink RNA Mini Kit	Invitrogen	Cat # 12183018A
0.45 µm Acrodisc Syringe Filters	Pall	Cat # 4614
**Recombinant DNA**		
pLenti Guide Puro	Addgene	Plasmid #52963
pENTR1a	Addgene	Plasmid #17451
pLenti CMV blast DEST	Addgene	Plasmid #10462
pLenti CMV no selection marker	This paper	
pLKO.1 puro pLenti CMV no selection marker	This paper	
pLKO.1 puro	Addgene	Plasmid #8453
**Software and websites**		
Prism v9.0	GraphPad	
Rstudio	Posit	https://posit.co/download/rstudio-desktop/
Synthego guide design tool	Synthego	https://design.synthego.com
TIDE analysis		https://tide.nki.nl/
**Other**		
Glomax Explorer	Promega	
CFX Connect Real-Time System	BIO-RAD	
Whole-body exposure chamber 099C A4212	Glas-Col	
Odyssey CLx	LI-COR	

**Table:** 

Primer and guide sequences		
qPCR*_IFNβ* Human F	This paper	CTTCTCCACTACAGCTCTTTCC
qPCR*_IFNβ* Human R	This paper	GCCAGGAGGTTCTCAACAATA
qPCR*_IFIT1* Human F	This paper	CCAGAAATAGACTGTGAGGAAGG
qPCR*_IFIT1* Human R	This paper	CCCTATCTGGTGATGCAGTAAG
qPCR*_β-ACTIN* Human F	This paper	GACCACCTTCAACTCCATCAT
qPCR*_β-ACTIN* Human R	This paper	CCTGCTTGCTAATCCACATCT
ms_*Actin*_qpcr_FW	This paper	GGTGTGATGGTGGGAATGG
ms_*Actin*_qpcr_RV	This paper	GCCCTCGTCACCCACATAGGA
ms_*Ifnβ*_qPCR_FW	This paper	TCCGAGCAGAGATCTTCAGGAA
ms_*Ifnβ*_qPCR_RV	This paper	TGCAACCACCACTCATTCTGAG
ms_*Ifit1*_qPCR_FW	This paper	CGTAGCCTATCGCCAAGATTTA
ms_*Ifit1*_qPCR_RV	This paper	AGCTTTAGGGCAAGGAGAAC
hu_*Cbl*_qPCR_Fw	This paper	GGTTGTCTCTGGATGGTGATC
hu_*Cbl*_qPCR_Rv	This paper	GACCACTACCTTGCTGACAG
hu_*Tlr8*_qPCR_Fw	This paper	TCCTGCATAGAGGGTACCATTCTG
hu_*Tlr8*_qPCR_Rv	This paper	TCGGCGCATAACTCACAGGAAC
hu_*Cxcl10*_qPCR_Fw	This paper	CTGAAAGCAGTTAGCAAGGAAAGGTC
hu_*Cxcl10*_qPCR_Rv	This paper	GTAGGGAAGTGATGGGAGAGGC
hu_shRNA_Cbl_Fw	This paper	CCGGGCCGATGTGAAATTAAAGGTACTCGAGTACCTTTAATTTCACATCGGCTTTTTG
hu_shRNA_Cbl_Rv	This paper	AATTCAAAAAGCCGATGTGAAATTAAAGGTACTCGAGTACCTTTAATTTCACATCGGC
hCBL_C > A_Fw	This paper	GATGGGCTCCACATTCCAACTAGCTAAAATATGTGCTGAAAATGATAAGGATGTAAAG
hCBL_C > A_Rv	This paper	CTTTACATCCTTATCATTTTCAGCACATATTTTAGCTAGTTGGAATGTGGAGCCCATC
hCBL_shRNAMut_Fw	This paper	AGGTCAGGGCTGTCCTTTCTGTAGGTGCGAGATTAAAGGTACTGAACCCATCGTGGT
hCBL_shRNAMut_Rv	This paper	ACCACGATGGGTTCAGTACCTTTAATCTCGCACCTACAGAAAGGACAGCCCTGACCT
Lg_mCbl_gRNA_Fw	This paper	CTTGTGGAAAGGACGACGTCTCTCACCGATCTTCTTGTCCACCGTGCAGTTTAAGAGCTATGCTGGAAACAG
Lg_ Scram_gRNA_Fw	This paper	CTTGTGGAAAGGACGACGTCTCTCACCGGCACTACCAGAGCTAACTCAGTTTAAGAGCTATGCTGGAAACAG
Lg_Scram_gRNA_Rv	This paper	AGTTACGCGTCTCTAAACTGAGTTAGCTCTGGTAGTGCGGGAAAGAGTGGTCTCATACAG

### Cell lines

THP-1 human monocytes (ATCC TIB-202) and HL-60 promyeloblast cells (ATCC CCL-240) were purchased from ATCC. THP-1 cells were cultured in RPMI media containing 10% fetal bovine serum (FBS), 10 mM HEPES, 1mM sodium pyruvate, 4500 mg/L glucose, and 0.05 mM 2-mercaptoethanol. HL-60 cells were cultured in RPMI containing 10% bovine growth serum (BGS), 15% fetal bovine serum (FBS), 1mM sodium pyruvate, 2 mM L-glutamine, and 1X MEM non-essential amino acids solution. HOXB8-ER and Cas9-expressing conditionally immortalized macrophages (CIMs) were cultured in RPMI media containing 10% FBS, 10mM HEPES, 1mM sodium pyruvate, 2mM L-glutamine, 1.5μl 2-mercaptoethanol, 2% GM-CSF as conditioned media from B16 murine melanoma cells, and 2 μM β-estradiol as previously described [55]. THP-1 and HL-60 cells were differentiated with DMEM supplemented with 10% FBS, 1mM pyruvate, 2 mM L-glutamine, 10 ng/ml phorbol 12-myristate 13-acetate (PMA), and 0.1 ng/ml 1,25 dihydroxy-vitamin D. Cells were differentiated for 72 h before experiments. CIM cells were differentiated by washing to remove β-estradiol and then cultured with DMEM supplemented with 10% FBS, 2 mM L-glutamine, 1 mM sodium pyruvate, 10% MCSF (derived from 3T3 MCSF conditioned media) for 7 days prior to experiments.

All cell lines generated using lentiviral vectors followed the same steps of transfection of HEK 293T Lenti-X cells with the transfer vector expressing the gene of interest, alongside the packaging plasmids psPAX2 and the envelope plasmid VSV-G. Viral supernatant was harvested 48 hours after transfection and either filtered with a 0.45 µm syringe filter and, for CIM cell transduction, concentrated using a PEG-8000 solution [71]. For transduction, viral supernatant with Polybrene 8 μg/ml was added for 12 h to THP-1 and HL-60 cells. CIM cells were transduced through spinfection and spun for 2h at 1000 RPM at 32° C and then incubated for 6 h before washing and culture in fresh media. Selective antibiotics were as follows: THP-1 and HL-60 cells were selected with 0.5 μg/ml of Puromycin or 4 μg/ml of Blasticidin. CIM cells were selected with 8 μg/ml of Puromycin or 4 μg/ml of Blasticidin.

### Bacterial strains

*Mtb* strains were cultured in 7H9 (BD) supplemented with 0.5% glycerol, 10% oleic acid-albumin-dextrose-catalase (OADC, Middlebrook), and 0.1% Tween-80 in bottles gently rotated at 40 RPM at 37° C or on 7H10 plates supplemented with 10% OADC, 0.5% glycerol, and 0.1% Tween 80 at 37°C. The *lpqN* mutant has been previously analyzed to confirm the location of the Himar 1 Tn insertion disrupting the *lpqN* (*Rv0583c*) locus and complementation by wild-type *lpqN* [45]. PDIM synthesis was confirmed for both the parental CDC1551 strain and the lpqN mutant by extracting apolar lipids with petroleum ether, and resolving them on C18 TLC with a mobile phase of petroleum ether:acetone (98:2). Lipids were visualized by staining in 0.2% Amido Black B in 1 M NaCl followed by destaining in 20% methanol (45 and S4 Fig).

### Macrophage infection for RNA harvest

*Mtb* strains were prepared for inoculation by washing twice in PBS with 10% heat-inactivated horse serum, gently sonicated, and spun at 500 rpm for 5 min. to generate a fine bacterial suspension. Bacteria were opsonized in 10% heat-inactivated horse serum and macrophages were infected at an MOI = 10 for 6 h by spinfection at 1200 RPM for 10 minutes followed by washing in PBS to achieve an infection rate of ~90%. After incubation, wells were washed once with 1X PBS and harvested in Trizol for RNA extraction.

### Macrophage infection for lux assay

For luminescent growth assays, cells were plated in triplicate in 96 well plates at a density of 8 x 10^4^ cells per well for THP-1 cells and 1.5 x 10^5^ cells per well for CIM cells. Macrophage cell density at the time of infection was carefully matched between control and experimental cell lines to within <15% variance by plating sets of control wells across a gradient of densities and using SYBR gold to quantify nucleic acid content of plated experimental and control cells. Macrophages were infected at an MOI = 0.8 to achieve an infection rate of ~50%, and after spinfection plates are incubated for 30 minutes at 37°C. After the incubation, the monolayers were washed with PBS containing 1% heat-inactivated horse serum twice, and bacterial luminescence was measured over time in a GloMax Explorer warmed to 37 degrees C. Macrophage growth media was changed every other day. Colony-forming units (CFUs) were quantified by lysing triplicate wells of macrophages in sterile water and plated in serial dilutions on 7H10 agar supplemented with OADC and 0.1% Tween-80. Plates were incubated at 37° C for 3 weeks prior to CFU enumeration.

### Immunofluorescence microscopy

Coverslips were coated with poly-L lysine for 1h at 37-deg and macrophages seeded at 3x10^5^ cells per coverslip. Cells were infected with *lpqN Mtb* at an MOI of 1. After 96 h cells were fixed for 20 minutes in 4% PFA, permeabilized in 0.1% Triton X-100 and incubated with Rhodamine B for 15 minutes at 37-deg to stain bacteria. After washing cells were stained with antibody recognizing ATP6E (1:100, 3h), followed by an anti-rabbit 647 secondary antibody (1:1,000, 1 h). Images were then acquired on a Thermo Evos FL Auto 2 microscope. For ATP6E localization an investigator blinded to sample identity scored each phagosome as positive or negative for colocalization. For lysosome staining with acidotropic dye the *lpqN* mutant was labeled with Cell Trace Far Red at 1:100 dilution for 30 minutes at 37°C prior to infection. Cells were infected for 5 days and then exposed to CytoFix Red Lysosomal Stain for 60 minutes at 37°C prior to PFA fixation. Cells were then imaged and phagosomes scored as above. For bacterial MFI measurements, phagosomes were analyzed using ImageJ software, with phagosomes systematically identified by the ImageJ Particle Analysis plug-in, and then quantified, also using the automated Particle Analysis plug-in. The Particle Analysis plug-in was used to systematically identify objects (bacterial phagosomes) and calculate the intensity of each pixel. Background intensity was determined on each slide by selecting a region between cells with no staining and this background value was subtracted from each pixel. The plug-in was then used to sum the pixel intensities to calculate the total pixel intensity for each object.

### Western blots

Protein in cell lysates was quantified by BCA. 30–50 μg of protein lysate was separated by SDS-PAGE and transferred onto nitrocellulose membranes and blocked with LI-COR blocking buffer at 0.5x for 1h. Primary antibodies were used at varying dilutions and incubated 1.5 h at room-temperature or, in the case of anti-CBL antibody, 4°C overnight. Secondary antibodies were used at 1:10,000 dilution for 1h. After probing for indicated antibodies, the membranes were imaged on an Odyssey scanner (LI-COR) and quantified in ImageJ using integrated fluorescent intensity with background subtraction.

### RNA purification

Nucleic acid-stimulated macrophages were lysed in PureLink lysis buffer per manufacturer instructions. *Mtb-* infected macrophages were lysed in Trizol. In both cases, lysates were purified with silica spin columns (Purelink) per manufacturer instructions.

### Macrophage nucleic acid stimulation and RT-qPCR

Differentiated THP-1 and HL-60 cells were plated at 8 x 10^5^ per well in a 12-well plate. Cells were stimulated with 10 ng of RNA or 20 ng of DNA delivered by Lipofectamine 2000 as per manufacturer instructions and incubated for 6 h. After RNA purification, cDNA was generated using 500ng total RNA with Maxima H minus reverse transcriptase and diluted 1:10 before qPCR analysis with the indicated primers and SYBR Green I detection of products.

### RNA-seq

Differentiated THP-1 cells were infected at MOI = 10 for 6 h with *lpqN Mtb* in three independent experiments on different days. RNA was purified and barcoded 3’Tag-Seq libraries prepared using the QuantSeq FWD kit (Lexogen, Vienna, Austria) for multiplexed sequencing according to the recommendations of the manufacturer by the UC Davis DNA Sequencing core. The fragment size distribution of the libraries was verified via micro-capillary gel electrophoresis on a Bioanalyzer 2100 (Agilent, Santa Clara, CA). The libraries were quantified by fluorometry on a Qubit instrument (Life Technologies, Carlsbad, CA), and pooled in equimolar ratios. Twelve libraries were sequenced on one lane of an Aviti sequencer (Element Biosciences, San Diego, CA) with single-end 100 bp reads. The sequencing generated more than 4 million reads per library. Analysis of human data: HTStream was used to remove PhiX, adapter sequences, polyA tails, low quality sequences from end of reads, N bases, and reads less than 50 base pairs. After preprocessing, STAR was used to align reads to GRCh38.p13 human genome. UMI-tools was used to remove PCR duplicates post-alignment. Next, feature Counts was used to count reads whose alignment overlapped with genes using the gencode genome corresponding to the human genome version used. Annotation used was version 41. R was used for limma-voom pipeline on R with multiple testing correction using Benjamini-Hochberg procedure.

### Plasmid construction for short shRNA and sgRNA delivery

shRNA expression plasmids were constructed by digesting pLKO.1 cloning vector with AgeI and EcoRI and annealed to oligonucleotides containing the 21 nucleotide shRNA encoding sequence. For sgRNA, 20 nucleotide targeting sequences were designed to target regions in the first exon of each locus using the Synthego design tool. The targeting sequence was then fused by overlap-extension PCR to a second-generation tracRNA [85] and inserted into the HpaI and XhoI sites of pSicoR. The dual-guide sgRNA vector was constructed using a synthesized gene block containing the two guides of interest and an H1 promoter from Integrated DNA technologies (IDT) that were digested with BsmBI v2 on both ends and ligated to BsmBI v2 digested Lentiguide Puro vector (Addgene) with a second-generation tracRNA [86]. Evaluation of editing efficiency was performed using TIDE.

### CRISPR/Cas9 genome editing

After lentiviral delivery of sgRNA, cells were selected for 1 week with puromycin. The genome editing efficiency of the polyclonal population was evaluated by PCR amplification of the targeted exon followed by Sanger sequencing and TIDE analysis. The polyclonal population was used for experiments.

### Site-directed mutagenesis

The wild-type and ligase mutant copies of CBL for both THP-1 and CIM cells contain synonymous mutations at the targeting sites of either the RNAi or CAS9 system to prevent degradation. Site-directed mutagenesis was carried out to generate catalytically inactive CBL mutants using pENTR1a plasmids (Addgene). Mutagenesis was validated through Sanger sequencing. Following validation, gateway cloning was used to transfer the mutated CBL open reading frame from pENTR1a into lentiviral pDEST vectors (Addgene). For THP-1 cells, constructs were cloned into a pLenti CMV Blast vector (Addgene). For CIM cells, constructs were fused by overlap extension PCR directly to a T2A peptide and Blasticidin resistance gene and were cloned into pLenti CMV modified to remove other antibiotic resistance genes [87].

### di-Gly Enrichment

3 x 10^7^ cells were plated 24 h before infection. Cells were infected with *lpqN Mtb* at an MOI = 10 for 6 h. 2 h prior to harvest, 1 μM of Bortezomib was added. For harvest, cells were washed 1X with PBS then scraped in ice-cold methanol+ 0.1 M glycine pH 2.5 to precipitate protein, and inactivate proteases and *Mtb.* Cells were then removed from the BSL3. Chloroform was added to 20% volume to assist precipitation, incubated on ice for 10 minutes and samples centrifuged to pellet protein. The pellets were washed once with ice-cold methanol, recentrifuged, and dried. Protein was resolubilized in 9M Urea 25 mM HEPES pH 8.5, 1 mM chloroacetamide (CAM), 0.5x Complete-mini protease inhibitor, and 0.5 mM EDTA with sonication. Proteins were then reduced for 45 minutes with 5 mM dithiothreitol (DTT) at room temperature and alkylated with 20mM CAM for 20 minutes at room temperature and in the dark. BCA assay was performed, samples were diluted 1:4 in HEPES pH 8.0, and 2.5 mg of protein was digested with Lys-C at enzyme to substrate ratio 1:100 at 37°C for 2 h. Subsequently, samples were digested overnight at 37° C with trypsin enzyme to substrate ratio 1:100. Digestion was stopped with 0.5% TFA and then incubated on ice for 15 minutes to allow urea to precipitate and samples centrifuged to pellet debris. Peptides were desalted with 500 mg Sep-Pak C18 columns as per manufacturer instructions and lyophilized.

2.5 mg of lyophilized peptides were resuspended with 0.5x immunoaffinity purification (IAP) buffer (50mM MOPS, pH7.2; 10 mM sodium phosphate; 50 mM NaCl) and sonicated. 10 μl cross-linked anti-K-ε-GG antibody beads were washed 3x in PBS. Samples were then mixed with 10 μl cross-linked anti-K-ε-GG antibody beads and incubated at 4°C for 2h. The peptide-bead mixture was then washed four times with PBS and one time with 0.1x IAP. Peptides were eluted with 100 μl 0.15% TFA and analyzed by LC-MS/MS with on-tip clean up prior to MS run.

### Mass spectrometry

Chromatography was performed on an Evosep 1 using either the 60 spd (di-Gly) or 30 spd (total protein) method. Each sample was loaded onto a disposable Evotip C18 trap column (Evosep Biosystems, Denmark) as per the manufacturer’s instructions. Briefly, Evotips were wetted with 2-propanol, equilibrated with 0.1% formic acid, and then samples were loaded using centrifugal force at 1200g. Evotips were subsequently washed with 0.1% formic acid, and then 200 μL of 0.1% formic acid was added to each tip to prevent drying. The tipped samples were subjected to nanoLC on a Evosep One instrument (Evosep Biosystems). Tips were eluted directly onto a PepSep analytical column, dimensions: 150umx10 cm C18 column (PepSep, Denmark) with 1.5 μm particle size (100 Å pores) (Bruker Daltonics), and a ZDV spray emitter (Bruker Daltonics). Mobile phases A and B were 100% water with 0.1% formic acid (v/v) and 100% Acetonitrile 0.1% formic acid (v/v), respectively. The standard pre-set method of 30 samples-per-day was used, which is a 44-minute run or 60 spd which is a 21-minute run.

Mass Spectrometry – Performed on a hybrid trapped ion mobility spectrometry-quadrupole time of flight mass spectrometer (timsTOF Pro 2 and timsTOF HT, (Bruker Daltonics, Bremen, Germany) with a modified nano-electrospray ion source (CaptiveSpray, Bruker Daltonics). In the experiments described here, the mass spectrometer was operated in diaPASEF mode. Desolvated ions entered the vacuum region through the glass capillary and deflected into the TIMS tunnel which is electrically separated into two parts (dual TIMS). Here, the first region is operated as an ion accumulation trap that primarily stores all ions entering the mass spectrometer, while the second part performs trapped ion mobility analysis.

DIA PASEF: The dual TIMS analyzer was operated at a fixed duty cycle close to 100% using equal accumulation and ramp times of 75 ms each. For the 30spd Evosep run, Data-independent analysis (DIA) scheme consisted of one MS scan followed by MS/MS scans taken with 6x3 = 18 precursor windows at width of 50Th per 6x3 = 560 ms cycle, over the mass range 8x3 = 300–1200 Dalton. The TIMS scans layer the doubly and triply charged peptides over an ion mobility -1/k0- range of 0.707-1.29 V*sec/cm2. The collision energy was ramped linearly as a function of the mobility from 59 eV at 1/K0 = 1.2 to 20 eV at 1/K0 = 0.7. For the 60spd Evosep run, Data-independent analysis (DIA) scheme consisted of one MS scan followed by MS/MS scans taken with 5x7 = 35 precursor windows at width of 30^Th^ = 763 ms cycle time over the mass range 286–1307 Dalton. The TIMS scans layer the doubly and triply charged peptides over an ion mobility -1/k0- range of 0.7-1.3 V*sec/cm2. The collision energy was ramped linearly as a function of the mobility from 59 eV at 1/K0 = 1.2 to 20 eV at 1/K0 = 0.

### Data analysis

LC-MS files were processed with Spectronaut version 18.4 (Biognosys, Zurich, Switzerland) using DirectDIA analysis mode. Mass tolerance/accuracy for precursor and fragment identification was set to default settings. The unreviewed FASTA for Mus Musculus, UP000000589, downloaded from UniProt and a universal library of common laboratory contaminants (Frankenfield et al, 2022). For di-Gly analysis, a maximum of two missing cleavages were allowed, the required minimum peptide sequence length was 7 amino acids, and the peptide mass was limited to a maximum of 4600 Da. Carbamidomethylation of cysteine residues was set as a fixed modification, and methionine oxidation, acetylation of protein N termini and ubiquitination as variable modifications. A decoy false discovery rate (FDR) at less than 1% for peptide spectrum matches and protein group identifications was used for spectra filtering (Spectronaut default). Decoy database hits, proteins identified as potential contaminants, and proteins identified exclusively by one site modification were excluded from further analysis. For total protein: DIA data was analyzed using Spectronaut 18 using the direct DIA workflow with PTM localization selected. Trypsin/P Specific was set for the enzyme allowing two missed cleavages. Fixed Modifications were set for Carbamidomethyl, and variable modifications were set to Acetyl (Protein N-term), Oxidation, and ubiquitination. For DIA search identification, PSM and Protein Group FDR were set at 0.01%. Mass spectrometry statistical analysis was performed in Spectronaut (Biognosys) using t-test with multiple testing correction.

### Pathway enrichment and ISG overlap analysis

Functional and pathway enrichment analysis for RNA-seq and ubiquitination proteomics were done on the set of genes with FDR <= 0.05 and log_2_ fold-change >= 0.5 using the DAVID database (Database for Annotation, Visualization and Integrated Discovery; https://david.ncifcrf.gov/) [88]. The significantly enriched biological pathways were identified with FDR < 0.05. The pathways were visualized using R. To identify CBL-dependent genes that are also known ISGs we determined the overlap between our dataset and the established set of type I IFN ISGs from the Interferome Database [46].

### Statistics

Statistical significance for RT-qPCR data was performed using GraphPad Prism version 10 (GraphPad Software, LLC) using a two-tailed t-test when comparing paired data. Statistical analysis for RNA-sequencing datasets was performed using the limma-voom pipeline on R with multiple testing corrections using Benjamini-Hochberg procedure. Ubiquitination statistical analysis was carried out within Spectronaut (Biognosys) using the integrated statistical package with randomized imputation of values near the limit of detection if a peptide was undetectable in a particular sample and then performing t-tests with multiple testing correction and FDR calculation. For microscopy, statistical analysis of colocalization was performed using paired t-test, and phagosome intensity distributions analyzed by non-parametric Wilcoxon rank-sum test.

## Supporting information

S1 Fig (A) CFU determination in THP-1 cells expressing either CBL-specific or non-targeting control shRNA 5 d after infection with *lpqN Mtb.* (B) Luminescent growth assay of *lpqN Mtb* in THP-1 cells depleted of CBL by a second independent shRNA. (C) RNA-seq analysis of control THP-1 cells expressing a non-targeting shRNA, comparing uninfected cells and *lpqN Mtb-*infected cells 6h after infection.
(TIF)

S2 Fig Luminescent growth assay of CBL-sensitive *lpqN Mtb* in the indicated DKO cell lines lacking *Cbl* and putative substrates with genes disrupted by CRISPR/Cas9 genome-editing. (TIFF)

S3 Fig Additional analysis of wild-type *Mtb* and *lpqN* mutant *Mtb* in *Cbl*^*-/-*^ and Faf2^*-/-*^ macrophages. A) Luminescent growth assay of wild-type *Mtb* in the indicated mutant CIM cells. B) *LpqN Mtb* was covalently labeled with Cell Trace and inoculated into CIM cells of the indicated genotype. 5 d post-infection, lysosomes were stained with acidotropic CytoFix Red Lysosomal Stain, and analyzed by microscopy to determine colocalization between bacteria and lysosomes. Arrowheads indicate *Mtb*-lysosome colocalization events. C) Quantification of colocalization between *Mtb*-containing phagosomes and acidified lysosomes.
(TIF)

S4 Fig A) Analysis of PDIM synthesis in *lpqN* mutant and wild-type *Mtb.* Apolar lipids were extracted, resolved by thin layer chromatography and visualized with Amido Black stain.
(TIF)

S1 Table RNA-Seq dataset. (XLSX)

S2 Table Di-Gly MS dataset. (XLSX)

S3 Table Total protein MS dataset. (XLSX)

S4 Table ISG overlap with CBL-regulated genes. (TXT)
