# Supplementary figures and images for: The ubiquitin ligase CBL and Fas-associated factor 2 cooperate to regulate the innate immune response to M. tuberculosis
Source: PLoS Pathog. 2026 Mar 17;22(3):e1013974. doi: 10.1371/journal.ppat.1013974 (PMC13089898; doi:10.1371/journal.ppat.1013974)

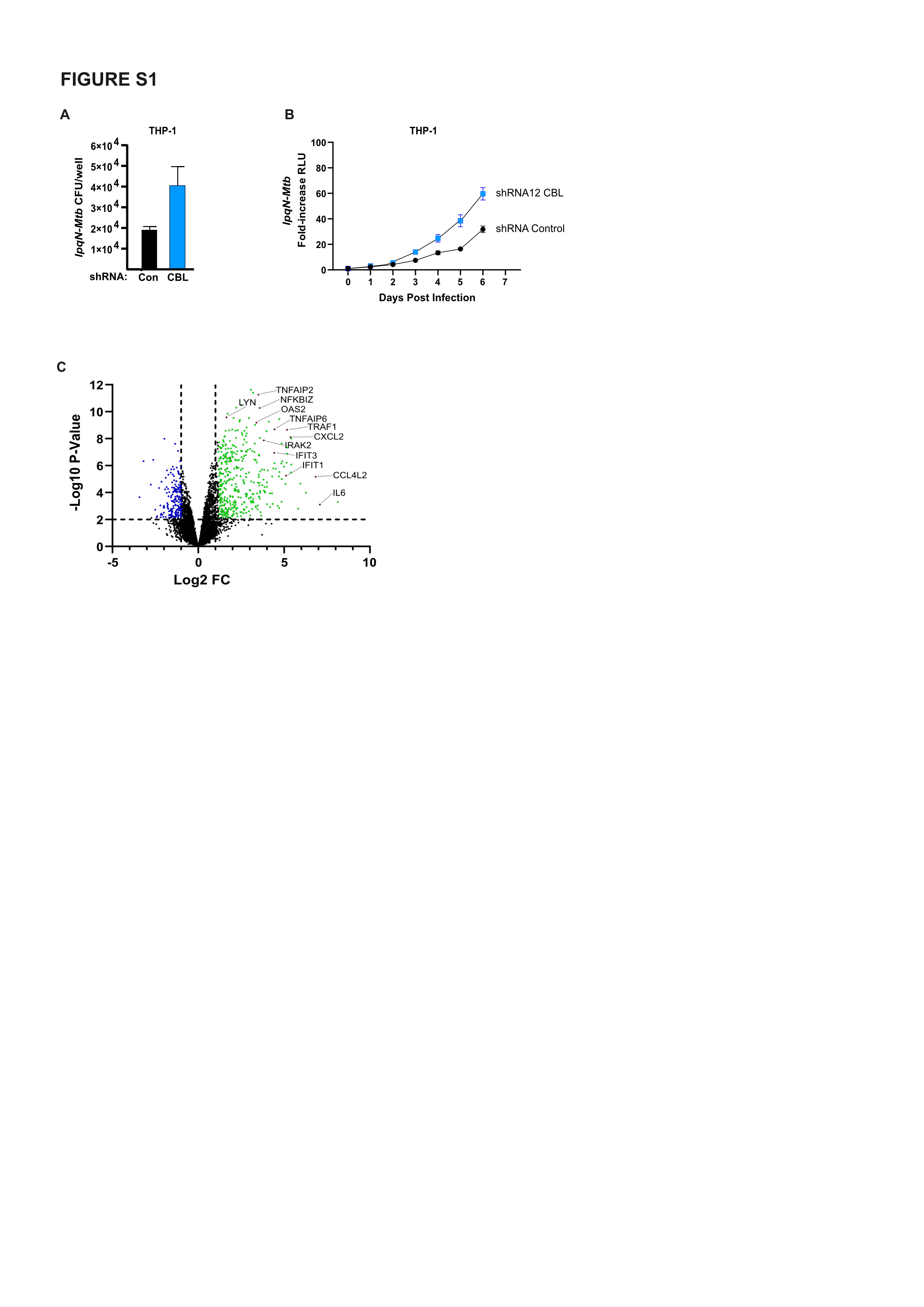

Supplement: S1 Fig — (B) Luminescent growth assay of lpqN Mtb in THP-1 cells depleted of CBL by a second independent shRNA. (C) RNA-seq analysis of control THP-1 cells expressing a non-targeting shRNA, comparing uninfected cells and lpqN Mtb-infected cells 6h after infection. (TIF) [file ppat.1013974.s001.tif]

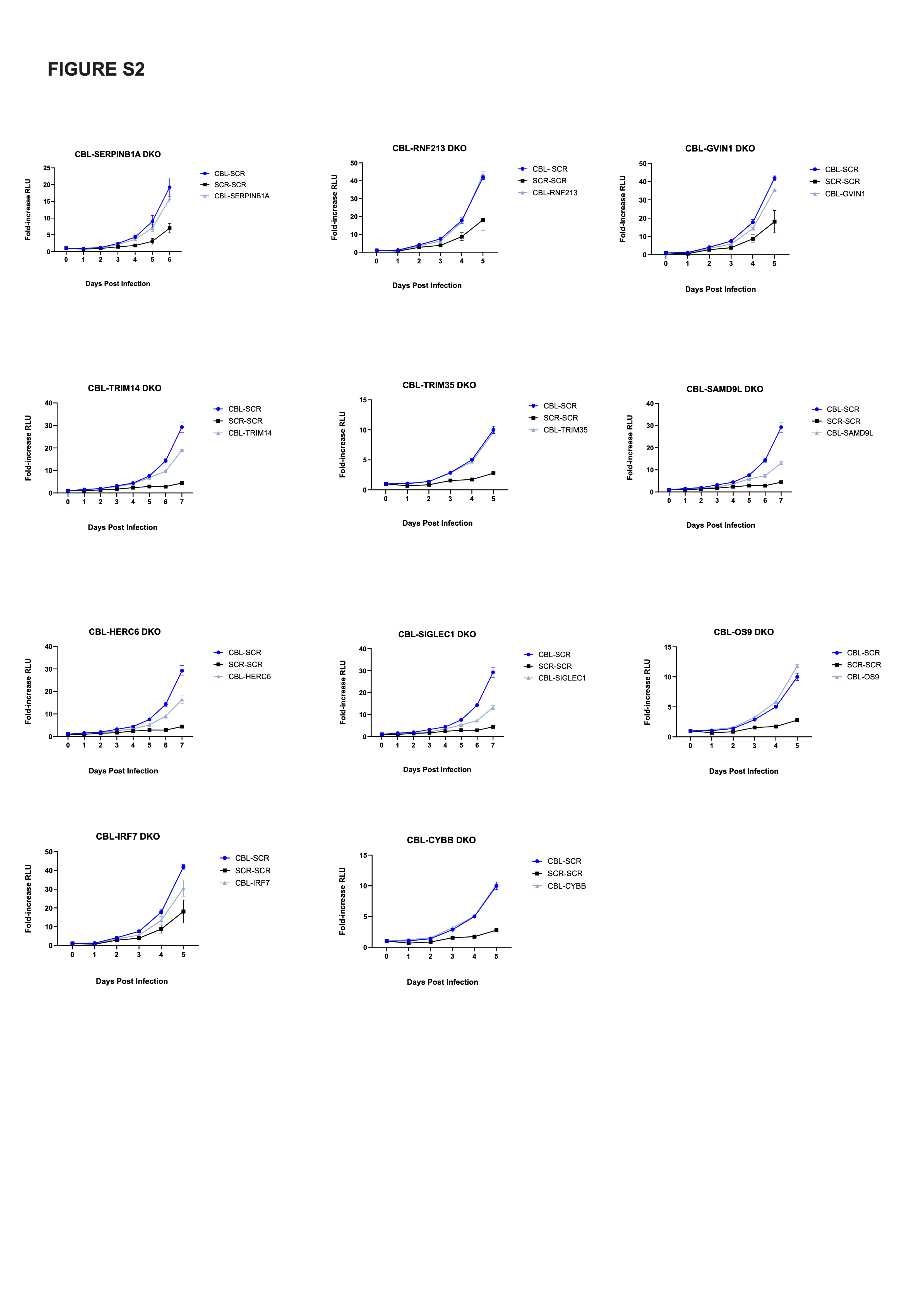

Supplement: S2 Fig — (TIFF) [file ppat.1013974.s002.tiff]

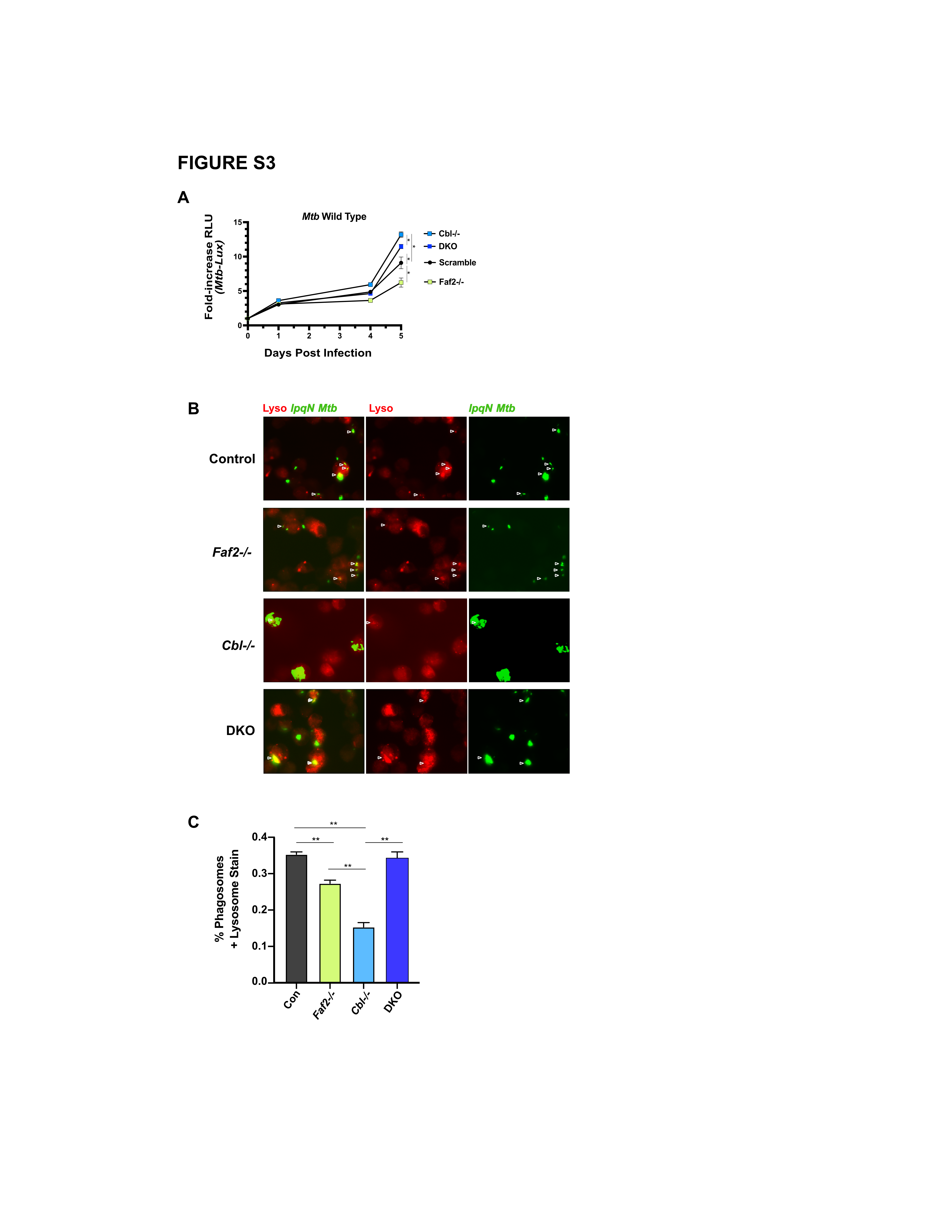

Supplement: S3 Fig — A) Luminescent growth assay of wild-type Mtb in the indicated mutant CIM cells. B) LpqN Mtb was covalently labeled with Cell Trace and inoculated into CIM cells of the indicated genotype. 5 d post-infection, lysosomes were stained with acidotropic CytoFix Red Lysosomal Stain, and analyzed by microscopy to determine colocalization between bacteria and lysosomes. Arrowheads indicate Mtb-lysosome colocalization events. C) Quantification of colocalization between Mtb-containing phagosomes and acidified lysosomes. (TIF) [file ppat.1013974.s003.tif]

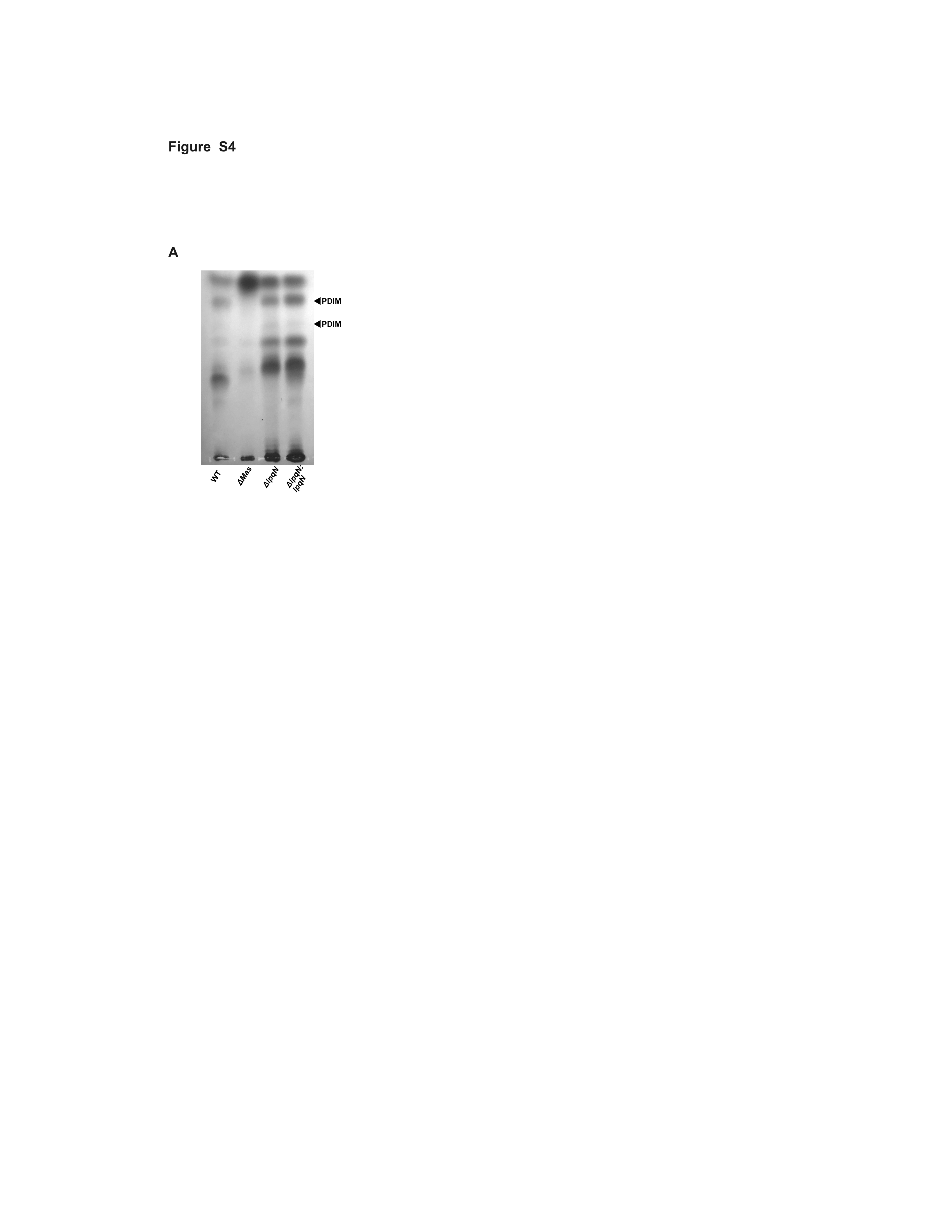

Supplement: S4 Fig — Apolar lipids were extracted, resolved by thin layer chromatography and visualized with Amido Black stain. (TIF) [file ppat.1013974.s004.tif]
